# Update on clinical research for food allergy treatment

**DOI:** 10.3389/falgy.2023.1154541

**Published:** 2023-07-14

**Authors:** Joshua Fowler, Jay Lieberman

**Affiliations:** Department of Pediatrics, Division of Pulmonology and Allergy/Immunology, University of Tennessee Health Science Center (UTHSC), Memphis, TN, United States

**Keywords:** food allergy, oral immunotherapy (OIT), biologic, sublingual immunotherapy (SLIT), epicutaneous immunotherapy (EPIT)

## Abstract

The incidence of IgE-mediated food allergy (FA) has continued to increase over the years which places substantial burden on patient health and quality of life. With no cure for this disease, the mainstay of management has been allergen avoidance. However, there have been advancements in FA treatment in recent years with multiple clinical trials utilizing novel and innovative therapeutic strategies. A landmark event came in 2020 with the first drug approval for food allergy with the approval of a product for peanut oral immunotherapy. In addition to oral immunotherapy, different delivery systems of immunotherapy (SLIT, EPIT) are being studied in addition to probiotics, biologic agents - used as monotherapy and as an adjunct, and modified allergens has taken place with the hope to further enhance existing therapeutic options. The hope through these continued developments is for therapies to emerge that will provide a more comprehensive benefit to this patient population.

## Introduction

Food allergy (FA) is an adverse health effect arising from a specific immune response that is reproducible following ingestion of a given food. Food allergy can be categorized into IgE-mediated and non–IgE-mediated, based on the pathophysiology of the reaction. In this mini-review we will be focusing on IgE-mediated FA and updates from recent trials and newer modes of treatment that are emerging. Food allergy affects roughly 8% of children and 10% of adults in western countries (1). There is no curative treatment for FA at this time, and the mainstay of management for most patients is allergen avoidance along with use of emergency should an accidental ingestion occur. Allergen avoidance necessitates dietary restrictions, with possible nutritional consequences, and can lead to food insecurity (2, 3). FA can also result in an impairment of quality of life and mental health of children and their families with some studies showing mothers of children with FA having higher state and trait anxiety scores than healthy controls (4, 5). FA can also cause financial burdens, related to both healthcare and ancillary costs, notably, from the need to spend more time finding alternative foods that are typically more expensive (6, 7). With prevalence increasing over the last couple of decades, the multifaceted burdens associated with food allergy and the negative effects on quality of life for patients and their families highlights the importance for the continued search for more specific treatment options (7).

Since the first randomized control trial (RCT) demonstrated efficacy in oral immunotherapy (OIT) over 20 years ago, numerous clinical trials have emerged looking into various approaches for the treatment of IgE-mediated FA. The most frequently assessed treatments are immunotherapy approaches, with OIT being used most frequently (8). Though there is now a regulatory approved OIT product available for peanut allergy in the United States and Europe, the standard of care for all other food allergens remains strict avoidance. While OIT has been effective, it does have limitations and novel forms of immunotherapy and other therapeutic approaches have shown promise (9). These methods include better tolerated and more effective preparations, dose, use of adjuvants, novel routes of immunotherapy and identifying patients most likely to benefit from immunotherapy. The two most studied alternative routes to OIT are sublingual (SLIT) and epicutaneous immunotherapy (EPIT). Multiple adjuvant agents have been tested in the context of improving benefit-risk ratio, from probiotics, to adjuvant medications (e.g., montelukast, antihistamines, and biologic treatments). There have also been studies looking into the efficacy of biologics as monotherapy. In the following text, we will summarize both the foundational clinical trials as well as highlight some of the more recent clinical trials that are underway for the different modes of treatment in food allergy.

## Immunotherapy

### Oral immunotherapy

Over the last 20 years there have been multiple clinical trials exploring different forms of IT, most notably for peanut, egg, and milk; and OIT remains the most well-studied form of immunotherapy for food allergy to date. No matter the food or the study, all OIT regimens involve the administration of gradually increasing amounts of allergenic protein up to a defined maintenance dose. While many foods have been studied, the largest amount of data available is for peanut (9, 10).

The PALISADE trial was a phase 3 international randomized placebo-controlled clinical trial evaluating AR101 and remains the largest peanut allergy immunotherapy study to date (10). Participants from ages 4 to 55 years old were screened by eliciting allergic dose-limiting symptoms at a challenge dose of 100 mg or less of peanut protein. The primary analysis of this trial in 496 patients aged 4–17 years indicated that 67.2% of AR101 - treated patients tolerated a single highest dose of at least 600 mg of peanut protein, whereas this dose was tolerated by only 4.0% of placebo-treated patients. This was determined during an exit food challenge which took place at around the 12-month period. The results from patients on OIT vs. placebo-treated patients, produced a between-group difference of 63.2% [95% confidence interval (CI), 53.0–73.3; *p* < 0.001]. Systemic allergic reactions and severe adverse events were observed in 14.2% and 6% of the active group, respectively, and 3.2% and 2% of the placebo group. These adverse events led to withdrawal from the study of 11.6% of the active group and 2.4% of the placebo group. The results of this study led to the first approved therapy for peanut allergy in the United States and Europe.

Many other studies of peanut OIT have been completed and all show similar types of efficacy depending on the patient population and the protocol selected (11, 12). Nurmatov et al. also conducted a systemic review and meta-analysis, comprising 31 studies and totaling over 1,200 participants, of which, 25 studies evaluated OIT for multiple foods including cow's milk, egg, peanut, and shellfish (13). A common finding is that adverse events are common, and in meta-analysis of available studies, anaphylaxis may actually be more common with OIT than with avoidance (12). Nurmatov et al. specifically concluded that OIT has demonstrated efficacy particularly in the pediatric population and that further study needs to be done exploring the role of OIT in the adult population as well as assessing the cost effectiveness and quality of life (13). Thus, while OIT can indeed lead to desensitization, its use is not without barriers (14).

**Figure 1:**
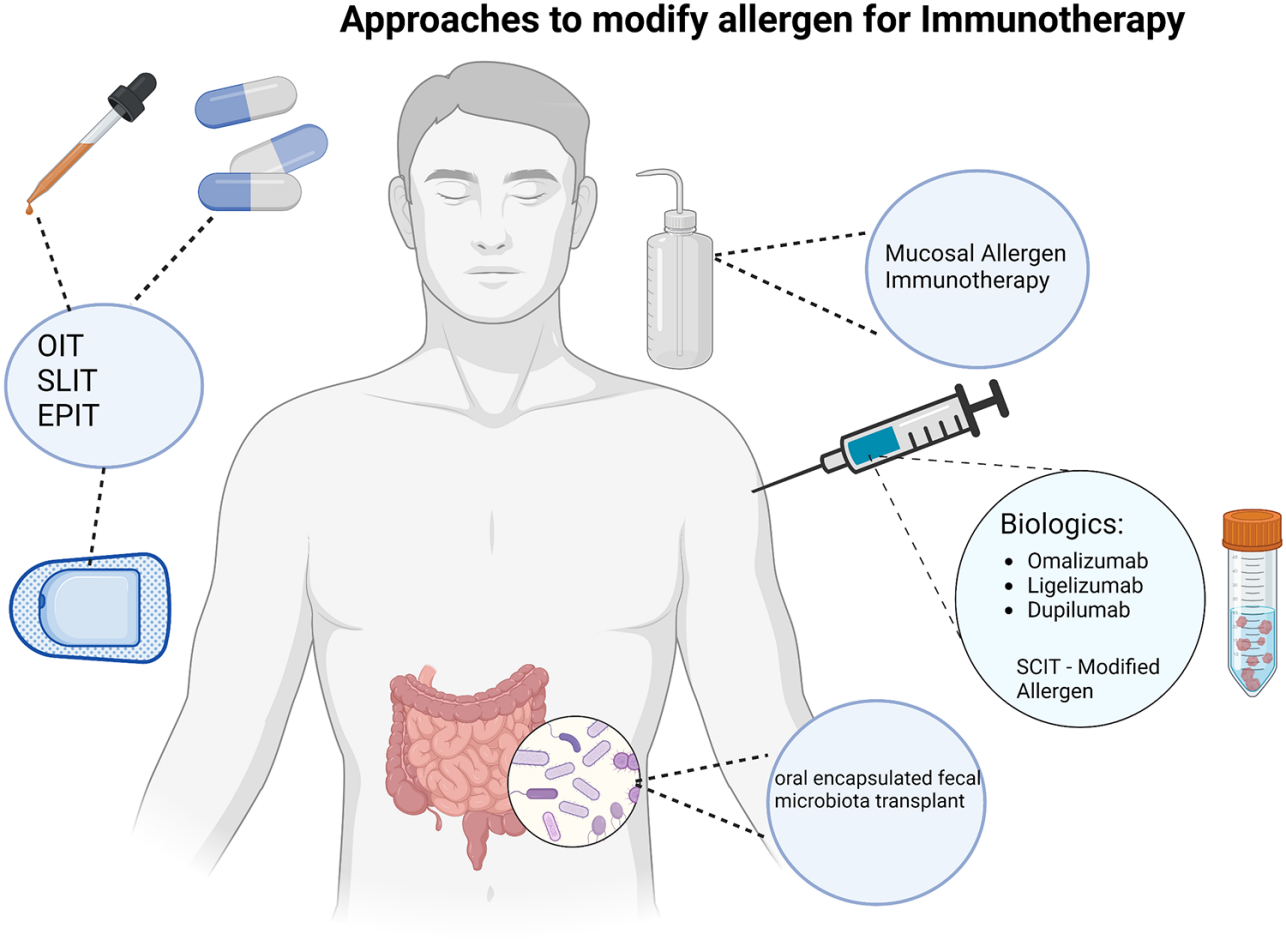
Approaches to modify allergen for Immunotherapy.

Due to the risk of adverse events associated with OIT, as well as to attempt to increase efficacy, groups have looked at various modifications to OIT. Modifications include starting OIT at an earlier age, adding adjuvants (e.g., probiotics) to the OIT (15, 16), adding pharmacotherapy prior to or in conjunction with the OIT (e.g., biologics or antihistamines) (17–19), and modifying the allergen itself (Figure 1). While a detailed discussion of some of these modifications is beyond the scope of this manuscript, some themes from these studies have surfaced. First, the earlier the OIT is started in life, it appears to be more effective. Peanut OIT has been done in infants successfully and efficacy rates are high in this patient population. This theme is further suggested by the fact that results of OIT in adults in the Palisade trial were not as effective as in children and the product did not gain approval for patients over 18 years of age. Second, addition of pharmacotherapy to the OIT regimen can aid in the adverse effects. For example, omalizumab has been shown to aid in decreasing adverse effects (17, 18). Oral antihistamines added to OIT has also been shown to mitigate some side effects such as oral and GI symptoms, but this did not affect patient quality of life (19). In addition, similar findings have been reported with probiotic use as an adjunct to OIT. Loke et al. has performed a multicenter, randomized phase 2b trial in children 1–10 years old with a confirmed peanut allergy, assessing efficacy and safety of adding a probiotic to OIT vs. OIT alone. Participants were randomized into either a peanut OIT and probiotic group, placebo probiotic and OIT group, or placebo probiotic and placebo OIT group. Participants received therapy for 18-month period and were reassessed at 12 months. Findings showed that 46% of participants in the probiotic/OIT arm and 51% of participants in the OIT only arm demonstrated sustained responsiveness compared to 5% of the placebo arm. Treatment-related adverse events were reported in 72 of 79 participants in the probiotic/OIT group and 73 of 83 participants in the OIT group. Exposure-adjusted incidence of adverse events was 10.58 in the probiotic/OIT group, 11.36 in the OIT, and 2.09 in the placebo group [OR: 0.92 (95% CI: 0.85–0.99) for PPOIT vs. OIT, *p* = 0.042; 4.98 (4.11–6.03) for PPOIT vs. placebo, *p* < 0.0001; 5.42 (4.48–6.56) for OIT vs. placebo, *p* < 0.0001], with differences seen in gastrointestinal symptoms and in children aged 1–5 years. These results show that the addition of a probiotic does not improve efficacy of OIT but could aid in the safety profile of OIT especially in pre-school aged children (15).

While the above studies have been carried out in peanut, OIT to other foods has similar efficacy, however there are fewer overall numbers of subjects receiving OIT with other foods and there is no approved pharmaceutical OIT product in any country to date. However, one must understand that OIT is allergen specific. Thus, OIT to peanut will only treat peanut allergy and will not desensitize to other food allergies. Due to this, some groups have looked at multi-food OIT (20–22). For example, Begin et al. performed a DBPCFC on 40 participants to prove food allergy, 15 of which were peanut allergic only and the other 25 participants had multiple food allergies; additional foods tested were tree nuts, sesame, dairy and egg. The study arms included peanut-allergic participants on Peanut OIT as well as participants with multiple food allergies on 5-Food OIT mix. The safety of single-food vs. multi-food OIT were similar in the study with rates of reaction per dose similar between the two groups (median of 3.3% and 3.7% in multi and single OIT group, respectively; *p* = .31). In addition, the severity of reactions was largely mild in both treatment groups, with abdominal pain being the most common complaint. Dose escalations proceeded similarly in both groups although, the participants in multiple food OIT group took longer to reach equivalent doses per food (median +4 mo.; *p* < .0001) (21). This data suggests that administering multiple food OIT simultaneously may be comparable to single food OIT in terms of efficacy and safety, though larger randomized control trials still need to be conducted. There is also a phase 1/2 randomized trial of a multi-food OIT product (NCT04856865), however no results have been published to date.

Another unknown regarding OIT is the optimal maintenance dose to target. Some studies have been conducted to assess this by comparing high vs. low dose OIT in terms of efficacy and safety profile. Takaoka et al. conducted a randomized control trial on 33 participants with an established milk allergy, assigning subjects in either a low dose, 20 ml, arm or a high dose, 100 ml arm. The target dose was reached during a rush escalation phase and was then maintained daily at home. The primary endpoint was to assess efficacy by a final OFC dose at 6 months of OIT. Adverse events were also evaluated while on OIT. Their results revealed a higher tolerated dose of milk protein in the final oral food challenge after being on OIT compared to before being on OIT, with no significant difference between final doses between the high dose and low dose (*p* = 0.767). Also of note, the high-dose group had significantly more adverse reactions than did the low-dose group during the maintenance phase (0.5%, 11/2,355 total events vs. 0.1%, 4/3,230 total events; *p* = 0.018) (23). These findings suggest low-dose OIT may offer similar efficacy and better safety than high-dose OIT, however the definition of “high-dose” and “low-dose” is not standardized currently and more research is needed in this regard.

Nowak-Wergryn et al. also performed a double-blind placebo-controlled clinical trial on vital wheat gluten (VWG) OIT on 46 subjects with wheat allergy. Participants were randomized 1:1 in either the low-dose VWG OIT arm or placebo arm, with biweekly escalation to 1,445 mg of wheat protein. After a year 1 DBPCFC, those in the low-dose VWG arm were continued on low-dose VWG OIT for another year and underwent a year 2 DBPCFC and, if passed, a subsequent off-therapy DBPCFC. Participants in the placebo arm were transitioned to high-dose VWG OIT, with a maximum dose of 2,748 mg. At the year one mark, 52.2% of the low-dose VWG OIT-treated and 0% of the placebo-treated subjects achieved the primary end point of a successfully consumed dose (SCD) of 4,443 mg of wheat protein or greater (*p* < .0001). At year two, 30.4% of the low-dose VWG OIT-treated subjects were desensitized to an SCD of 7,443 mg of wheat protein. Of the subjects in the placebo-treated arm who transitioned to the high-dose VWG OIT, 57.1% were desensitized after 1 year. No statistical significance was noted in successfully tolerated doses between those in the low-dose VWG OIT arm and in those who switched to high-dose VWG OIT. They concluded that about half of participants in this study were able to achieve desensitization after 1 year on both low-dose and high-dose VWG OIT (24). These results again support the idea that low-dose maintenance OIT seems to be as efficacious as high-dose OIT.

One final note on OIT for food allergies, is that the duration of therapy is currently unknown. There have been few long-term studies on alternate dosing regimens and rigorous trials examining results off therapy. Available data suggest that sustained unresponsiveness and possible “cure” may be possible in some patients, but no reliable predictors of response are available currently (25–27). In addition, some data on peanut OIT has shown that alternate dosing regimen (e.g., twice weekly dosing) once patients reach maintenance may be effective (28).

### Sublingual immunotherapy

The emergence of sublingual immunotherapy (SLIT) to be studied as a therapeutic option for food allergy came in part from the questions of safety profile and long-term efficacy that had come from clinical trials in OIT as well as the surprisingly high rate of systemic reactions seen with SCIT used for food allergy. To date, several studies of SLIT for IgE-mediated allergy have been conducted (29). Kim et al. were the first to conduct a double-blind, placebo-controlled study to evaluate the safety and efficacy of peanut SLIT (30). Results from their double-blind, placebo-controlled food challenge showed that the treatment group safely ingested 20 times more peanut protein than the placebo group (median, 1,710 vs. 85 mg; *p* = .011), and immunologic changes of immunotherapy (decrease peanut specific IgE levels and increase peanut-specific IgG4 levels) could be detected (30).

Since that first study, there have been other well-designed trials of SLIT (31–34). Nowak-Wegryzn et al. also performed a direct comparison of treatment protocols from multiple studies on SLIT and OIT. In this side-by-side comparison, maintenance and up-dosing were assessed as well as dosing interval, safety, and efficacy. Both SLIT and OIT require daily dosing, while SLIT does not require up-dosing and has lower dosage of daily maintenance, 2–7 mg compared to 300–5,000 mg for OIT (35). The overall theme from these studies (including a study comparing SLIT and OIT) is that SLIT can be efficacious in desensitizing to foods, but the level of desensitization appears to be to a lesser degree than to that seen with OIT, although SLIT appears to have an overall better safety profile (33, 35). Nowak-Wegryzn et al. highlighted that while OIT and SLIT differ significantly, there is likely a role for each modality depending on the target population and risk/benefit discussions. Further study into the role of adjuvant therapies for SLIT will also hopefully be of high yield, given the limited volume of food extract that can administered under the tongue (35).

Given the promise of SLIT, there are ongoing SLIT trial one of which will be conducted with a pharmaceutical grade product (NCT05440643).

### Epicutaneous immunotherapy

Epicutaneous immunotherapy (EPIT) is a more novel route of immunotherapy in which the allergen is delivered via a patch on the skin. The possible benefit of this route would be that very small amounts of allergen could be used for desensitization with fewer adverse effects as compared to OIT (36). This route was initially studied in pollen allergy (such as with OIT and SLIT), and has since been studied in well-designed trials for milk and peanut allergy (37–43). Results from these trials suggest that EPIT can lead to desensitization in some patients with IgE-mediated allergies. The robustness of the treatment effect appears to be more in line with SLIT than with OIT, but like SLIT, it is safer than OIT with fewer systemic reactions. Interestingly, like OIT, it appears that the earlier the EPIT is used, the more effective it is in achieving desensitization (40). However, low levels of desensitization may be enough to achieve goals of patients. This was demonstrated in studies for peanut EPIT where patients who were treated with EPIT for 12 months showed a relative risk reduction of 73.2%–78.4% when consuming peanut contaminated packaged food products (44, 45). In addition, there are data to suggest that the efficacy of EPIT improves the longer the therapy is used (41, 43). Unfortunately, to date, like OIT and SLIT, there is no reliable biomarker to accurately predict responders to EPIT. Clinical trials of EPIT are ongoing to attempt to better define efficacy in specific age ranges.

### Modified allergen immunotherapy and other routes

While subcutaneous immunotherapy (SCIT) is a common form of immunotherapy used for inhalant allergies, SCIT for peanut allergy proved to have too many adverse effects to pursue as a reasonable treatment option (46, 47). However, there are studies ongoing of SCIT with modified allergen such as with modified, aluminum hydroxide adsorbed peanut extract (NCT02991885). In addition, one groups is looking into mucosal allergen immunotherapy with the delivery mechanism of toothpaste (NCT04603300).

#### Biologic monotherapy

As outlined above, immunotherapy options for food allergy appear to be efficacious at achieving desensitization. However, there are some major drawbacks to these desensitization regimens, including adverse effects, the need for daily or near-daily therapy, lack of biomarkers that can predict success of therapy, and immunotherapy is antigen specific (i.e., treatment with one food would not desensitize to other foods if the patient is poly-allergic). Thus, other forms of treatment are desired. One form of therapy that has been most studied to date are biologics. As stated earlier, biologics have been used as adjuvants to OIT (17, 18), however, their use as a monotherapy may be more appealing. If efficacious, they would be allergen naïve, and allow for treatment of multiple allergies and likely have less adverse effects as compared to OIT.

Interestingly, the idea of using biologics to treat food allergy is not novel, and was studied as early 20 years ago when Leung et al. examined a humanized IgG1 monoclonal antibody against IgE called TNX-901 or Talizumab (48). That study showed that TNX-901 could raise the threshold of reactivity, similar to what the more recent OIT, SLIT, and EPIT trials showed. While TNX-901 was never developed further, the molecule is similar to omalizumab and ligelizumab. Both of these molecules are currently being studied in large phase III trials (NCT03881696, NCT04984876). In addition to these anti-IgE monoclonal antibodies, the anti IL4/IL13 receptor alpha monoclonal antibody dupilumab has also been studied (NCT03793608). Although results have not been published.

#### Other therapies

Various other pharmacologic agents are now being studied for IgE-mediated food allergies. For example, one study is planning on looking at the oral janus kinase (JAK) inhibitor abrocitinib for food allergy (NCT05069831), while another is evaluating the efficacy of the Bruton's tyrosine kinase (BTK) inhibitor remibrutinib for the treatment of food allergy (NCT05432388). Another group is looking at using infusions of nano-particle coated peanut protein (NCT05250856). There are no data on any of these therapies to date.

One final method of treatment being examined is using oral encapsulated fecal microbiota transplant in peanut allergic patients (NCT02960074), which does have some preliminary data (49).

## Conclusion

As the prevalence of food allergy continues to grow, the investigation for more complete treatment options for the most common food allergies remains a top priority for both clinicians and researchers. Many clinical trials have established both the efficacy and safety of OIT. From this groundwork, more clinical trials are underway to explore both different mechanisms of immunotherapy delivery as well as more novel therapeutic approaches. Each modality of treatment has been shown to offer benefit in certain patient populations and particular circumstances but not without long term challenges, whether that be adherence barriers or the potential negative effects on quality of life and the anxieties surrounding the safety profile of some of these therapeutic options. As more treatment approaches for the most prevalent food allergies continue to be explored, progress into the understanding of the interplay between efficacy, safety, and adherence in conjunction with a deeper knowledge of the potential long-term immunomodulator effects hope to usher in more comprehensive therapeutic options moving forward.
